# Highly Efficient and Stable Self‐Powered Mixed Tin‐Lead Perovskite Photodetector Used in Remote Wearable Health Monitoring Technology

**DOI:** 10.1002/advs.202205879

**Published:** 2022-12-09

**Authors:** Fengcai Liu, Kai Liu, Saqib Rafique, Zengyi Xu, Wenqing Niu, Xiaoguo Li, Yifan Wang, Liangliang Deng, Jiao Wang, Xiaofei Yue, Tao Li, Jun Wang, Paola Ayala, Chunxiao Cong, Yajie Qin, Anran Yu, Nan Chi, Yiqiang Zhan

**Affiliations:** ^1^ Center for Micro Nano Systems School of Information Science and Technology (SIST) Fudan University Shanghai 200433 P. R. China; ^2^ Key Laboratory for Information Science of Electromagnetic Waves (MoE) Department of Communication Science and Engineering Fudan University Shanghai 200433 P. R. China; ^3^ Key Laboratory of Micro and Nano Photonic Structures (MOE) and Shanghai Ultra‐precision Optical Manufacturing Engineering Research Center Department of Optical Science and Engineering Fudan University Shanghai 200433 P. R. China; ^4^ Faculty of Physics University of Vienna Vienna 1090 Austria; ^5^ Shanghai Frontier Base of Intelligent Optoelectronics and Perception Institute of Optoelectronics Fudan University 2005 Songhu Road Shanghai 200438 P. R. China

**Keywords:** functional passivating antioxidant strategy, flexible photodetectors, wearable health monitoring, wearable optical communication

## Abstract

Realization of remote wearable health monitoring (RWHM) technology for the flexible photodiodes is highly desirable in remote‐sensing healthcare systems used in space stations, oceans, and forecasting warning, which demands high external quantum efficiency (EQE) and detectivity in NIR region. Traditional inorganic photodetectors (PDs) are mechanically rigid and expensive while the widely reported solution‐processed mixed tin‐lead (MSP) perovskite photodetectors (PPDs) exhibit a trade‐off between EQE and detectivity in the NIR region. Herein, a novel functional passivating antioxidant (FPA) strategy has been introduced for the first time to simultaneously improve crystallization, restrain Sn^2+^ oxidization, and reduce defects in MSP perovskite films by multiple interactions between thiophene‐2‐carbohydrazide (TAH) molecules and cations/anions in MSP perovskite. The resultant solution‐processed rigid mixed Sn–Pb PPD simultaneously achieves high EQE (75.4% at 840 nm), detectivity (1.8 × 10^12^ Jones at 840 nm), ultrafast response time (*t*_rise_/*t*_fall_ = 94 ns/97 ns), and improved stability. This work also highlights the demonstration of the first flexible photodiode using MSP perovskite and FPA strategy with remarkably high EQE (75% at 840 nm), and operational stability. Most importantly, the RWHM is implemented for the first time in the PIN MSP perovskite photodiodes to remotely monitor the heart rate of humans at rest and after‐run conditions.

## Graphical abstract

A novel functional passivating antioxidant strategy is introduced to simultaneously improve crystallization, restrain Sn^2+^ oxidization, and reduce defects in mixed tin‐lead (MSP) perovskite films. This work highlights the first flexible photodiode using MSP perovskite with remarkable performance. Finally, the remote wearable health monitoring (RWHM) is implemented for the first time in the PIN MSP perovskite photodiodes to remotely monitor the heart rate of humans.

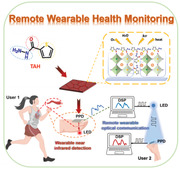

## 1 Introduction

Remote wearable health monitoring (RWHM) has been widely explored in recent years for forecasting warning, differential diagnosis, evaluation, rehabilitation training, and precision therapy of illness.^[^ ^1^, ^2^ ^]^ It also plays a vital role in better understanding the effect of special environments on physiological changes, such as space stations, ocean, and fire scenes.^[^ ^3^, ^4^ ^]^ In this context, wearable sensing devices (WSDs) not only offer a facile noninvasive method to gain vital signs indispensable for health monitoring but also can conveniently receive remote information by wireless real‐time visible light communication (VLC).^[^ ^2^, ^4^, ^5^ ^]^ One of the key WSDs are the flexible broadband photodiodes which play a key role in the resolution ratio, precision, and detection limits of the WSDs for RWHM systems.^[^ ^6^, ^7^ ^]^ The ideal broadband photodiodes for RWHM system should offer high sensitivity, detectivity, and stability at the operating bias.^[^ ^8^, ^9^ ^]^ The most commonly used RWHM wavelength ranges are between 600 and 1000 nm for vital signs monitoring and 400 and 500 nm for VLC. However, traditional high‐performance broadband photodetectors (PDs) are based on semiconductor materials such as silicon (Si), germanium (Ge), and indium gallium arsenide (InGaAs), which are rigid and need high temperature, vacuum, and unfriendly environmental manufacturing processes such as chemical vapor deposition, sputtering, and atomic layer deposition, leading to unrealizable flexible applications, large costs, and time‐consuming.^[^ ^10^ ^]^

In recent years, benefiting from the solution processes with low cost and excellent photoelectric properties, organic–inorganic hybrid perovskites have attracted extensive interests in photodetection.^[^ ^11^, ^12^, ^13^ ^]^ In addition, owing to the low‐temperature fabrication process, light‐weight and low Young's modulus,^[^ ^14^ ^]^ the flexible organic–inorganic hybrid perovskites devices have been extensively explored, revealing their great potential in wearable optoelectronics.^[^ ^13^, ^15^ ^]^ However, traditional pure lead perovskites are seriously harmful to the environment and the human health. Moreover, traditional pure lead perovskites and their integrated polymers and quantum dots variants still demonstrate inferior photons absorption and charge transfer in the near infrared (NIR) region, leading to low external quantum efficiency (EQE) in NIR region.^[^ ^16^ ^]^ Notably, mixed tin–lead hybrid perovskites not only are more environment‐friendly but also have a narrower bandgap than traditional pure‐lead hybrid perovskites, extending the high‐efficient responsivity to the NIR region, which have emerged as very competitive candidates in broadband PDs.^[^ ^8^, ^12^, ^17^, ^18^, ^19^ ^]^ However, there is a trade‐off between EQE and detectivity in mixed tin–lead (MSP) perovskite photodetectors (PPDs). While most of the recent researches only focused on the optimization of the upper and lower interfaces in the rigid MSP PPDs.^[^ ^10^, ^17^, ^19^, ^20^ ^]^ Although interface engineering can effectively reduce dark current, however it cannot achieve high EQE over 75% in the NIR region of the Sn–Pb PPDs without compromising other parameters. For example, Zhao et al.^[^ ^17^ ^]^ demonstrated Sn–Pb PDs with around 80% EQE in the NIR region using double‐sided interface engineering, but maximum detectivity is only 2.07 × 10^11^ Jones. Ollearo et al.^[^ ^8^ ^]^ reported Sn–Pb PD with ultralow dark current by replacing different electron‐blocking layers, achieving high detectivity (2.5 × 10^12^ Jones at 940 nm), but the EQE was 68% at 850 nm. Very recently, Najarian et al.^[^ ^21^ ^]^ reported a high EQE (85% in NIR region) and high detectivity (1.5 × 10^12^ Jones at 905 nm) simultaneously using disproportionation reaction of tin wire and NiO_X_ as hole transport layer. However, it required filtration of the tin wire from the perovskite solution before spin‐coating and removal of tin wire cannot effectively reduce Sn^4+^ in the subsequent procedures which ultimately compromises the stability. Therefore, it is highly desired to find functional passivating antioxidants (FPAs) possessing the high solubility to produce the reducing and passivating effect during and after the crystallization of mixed Sn–Pb perovskite films to minimize the defect density and maximize the EQE.

In this work, we report a FPA, thiophene‐2‐carbohydrazide (TAH), as a new FPA for simultaneous improvement in the EQE and detectivity of mixed Sn–Pb PPDs without removing the antioxidant during the material and device processing. The carbonyl and thienyl units (electron pair donor) in TAH molecule (the Lewis base) can interact with the undercoordinated Pb^2+^/Sn^2+^ by coordination bond and the FA^+^ by hydrogen bond in perovskite. While the hydrazine group in the TAH molecules can simultaneously reduce the oxidation of Sn^2+^ and interact with the I^−^ sites in the perovskite by hydrogen bonds. In summary, the suggested FPA provides a stronger perovskite surface‐passivator interaction through multiple interactions between the TAH molecules and both cations and anions in perovskite at the same time.^[^ ^22^ ^]^ Due to the interaction between perovskite and TAH, utilization of TAH can enhance perovskite grain size and restrain Sn^2+^ oxidation at the same time, enabling minimized defect density and maximized film absorption. Hence, a simultaneous increase in the detectivity and EQE exhibiting impressive values has been realized in this work. Owing to the fact that nonradiative recombination of charge carriers was remarkably suppressed, a high EQE in the NIR region (75.4% at 840 nm) along with the high detectivity (2.5 × 10^12^ Jones at 940 nm) and ultrafast response speed (*t*_rise_/*t*_fall_ = 94 ns/97 ns) have been achieved. While TAH‐treated Sn–Pb PPDs exhibited excellent stability under humidity and thermal stress due to the increased perovskite surface hydrophobicity along with the emergence of strong hydrogen and coordination bonding upon TAH passivation. To the best of our knowledge, it is one of the highest EQE and detectivity in the NIR region along with excellent stability, which is very rare to concurrently achieve for the Sn–Pb PPDs. Furthermore, one of the key highlights of this work is the flexible mixed Sn–Pb PPDs with high performance and stability which have been fabricated for the first time. Another remarkable achievement is to realize a wearable remote health monitoring application (health monitoring and optical communication integration application) that has been demonstrated for the first time with the flexible Sn–Pb PPDs as a broadband light receiver, which shines a light on the application in complex configuration with multiple functionalities.

## 2 Results and Discussion

### 2.1 Functional Passivating Antioxidants Strategy

**Figure 1:**
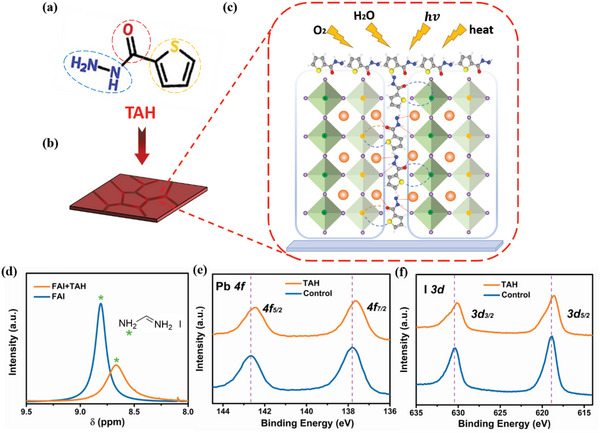
Functional passivating antioxidants strategy. a) The chemical structure of thiophene‐2‐carbohydrazide (TAH) molecule. b) Schematic representation of fabricated perovskite films with TAH molecule. c) Schematic showing the passivating mechanism between TAH and mixed Sn–Pb perovskite. d) ^1^H nuclear magnetic resonance (^1^H NMR) spectra of formamidinium iodide (FAI) films with/without TAH. e,f) X‐ray photoelectron spectroscope (XPS) spectra of Pb 4f orbit and I 3d orbit in control and TAH films.

As shown in **Figure** **1**a, the TAH molecule contains desired thienyl, carbonyl, and hydrazine multifunctional groups, which makes it ideal FPA material to realize high‐performance MSP PPDs. To explore the effect of TAH on the Sn–Pb films, two variants (with and without the TAH) of FA_0.7_MA_0.3_Pb_0.5_Sn_0.5_I_3_ (MA is methylammonium and FA is formamidinium) perovskite films (Figure 1b) were prepared from the precursor solutions using a one‐step method having antisolvent treatment (details in the Experimental Section). For simplicity, these two kinds of films hereafter will be named as “Control” and “TAH” samples. Stimulated by the fact that the multifunctional groups of TAH molecule can overcome the serious challenge for MSP PPDs, we envisioned that the TAH molecule as FPA in perovskite can easily interact with the undercoordinated Pb^2+^/Sn^2+^, I^−^ sites, and FA^+^ by coordination or hydrogen bond and mitigate the oxidation of Sn^2+^ by the reducing hydrazine group, which provides strong perovskite crystal grains surface‐passivator interaction as well as minimizes the oxidation, as shown in Figure 1c.

The role of TAH molecules was further elucidated using ^1^H nuclear magnetic resonance (^1^H NMR) and X‐ray photoelectron spectroscope (XPS). As depicted in Figure 1d (with the full regions shown in Figure S1, Supporting Information), the resonance signal at 8.81 ppm is attributed to the protonated ammonium in the formamidinium iodide (FAI) deuterated dimethyl sulfoxide (DMSO) solution.^[^ ^23^ ^]^ More interestingly, the ammonium signal shifted to low field (8.67 ppm) in mixed FAI and TAH deuterated‐DMSO solution. The shift indicates the formation of hydrogen bond between TAH and FAI, possibly resulted from strong electronegativity of O or N atoms in TAH and FA^+^.^[^ ^24^ ^]^ Due to the stronger electronegativity of O atom than the N atom, the hydrogen bonds are likely to be N−H^…^O=C.^[^ ^23^ ^]^ More the interaction between TAH and perovskite was characterized by using XPS measurement (Figure 1e,f and Figure S2, Supporting Information). Firstly, in comparison with control sample, the TAH sample exhibited S 2p peak, showing that TAH is present in perovskite films (Figure S2a, Supporting Information). This is in accordance with the fact that the TAH is neither soluble in the antisolvent nor it is volatile. Secondly, the TAH sample shows substantial peak shift compared with control sample, which suggests that a chemical bond between TAH molecule and perovskite has been formed. In detail, the characteristic spectra of Pb in the control sample show two peaks located at 142.66 and 137.83 eV, ascribed to the Pb 4f_5/2_ and Pb 4f_7/2_ orbits, respectively.^[^ ^25^ ^]^ However, the characteristic peaks of Pb in the TAH sample shifted to lower binding energies (142.44 and 137.62 eV) (Figure 1e). While there is an upshift in the characteristic peaks of Sn from 487.12 to 487.20 eV for Sn 3d_5/2_ orbit and from 495.60 to 495.65 eV for the Sn 3d_3/2_ orbit, as shown in Figure S2b (Supporting Information), this is attributed to Sn^2+^ which causes oxidation and easier loss of electrons.^[^ ^25^ ^]^ In addition, the characteristic peaks of I also shifted to lower binding energies from 630.42 to 630.22 eV and from 618.87 to 618.60 eV, showing the similar downshift with Pb peaks (Figure 1f).^[^ ^26^ ^]^ These downshifts may be attributed to the coordination bonding (C=O:Pb/Sn; S:Pb/Sn) between O or S atoms in TAH and undercoordinated Pb^2+^/Sn^2+^ in perovskite by Lewis base‐acid interaction as well as the another hydrogen bond (N−H^…^I) between hydrazine group (−NHNH_2_) and I^−^ in perovskite.^[^ ^27^, ^28^ ^]^ Finally, the characteristic peaks of N and O also shifted in TAH sample indicating there is a chemical state change of the FA^+^ in perovskite and C=O in TAH, which is consistent with the ^1^H NMR results and XPS peak shift of Pb^2+^/Sn^2+^(Figure S2c,d, Supporting Information). Based on these results, the specific interaction between TAH molecules and perovskite is authenticated and a schematic diagram of defect passivation mechanism for the TAH is shown in Figure 1c.

### 2.2 Effect of FPA Strategy on Mixed Perovskite Films

**Figure 2:**
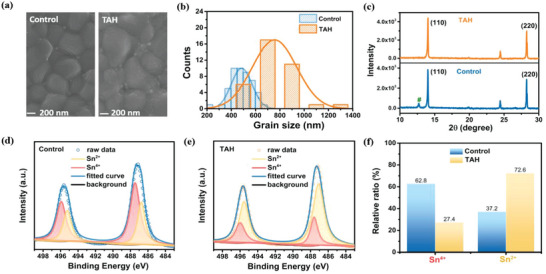
The effect of functional passivating antioxidant (FPA) strategy on mixed perovskite films. a) Top‐view scanning electron microscopy (SEM) images of control and thiophene‐2‐carbohydrazide (TAH) films. b) The statistical histograms of grain size in control and TAH films. c) X‐ray diffraction (XRD) patterns of control and TAH films. d,e) Sn 3d X‐ray photoelectron spectroscope (XPS) spectra of control and TAH films. f) The relative ratio of Sn^4+^ and Sn^2+^ in control and TAH films.

To further elucidate the effects of TAH on crystalline process and morphology of Sn–Pb perovskite films, more measurements on the homologous perovskite films were performed. Top‐view scanning electron microscopy (SEM) images and the corresponding statistics of grains size of control and TAH perovskite films are displayed in **Figure** **2**a,b and Figure S3 (Supporting Information). Compared to the control films, the TAH films show notably increased crystal grain size and more smooth morphology. The average grain size of control films is measured to be 477.69 nm, while it is 756.09 nm for TAH films. A similar result is found in the atomic force microscope (AFM) measurement, as depicted in Figure S4 (Supporting Information). After adding TAH in perovskite films, the surface roughness decreased and the crystal grain size increased. The larger grain size and more smooth morphology of the mixed Sn–Pb perovskite films suggest the grain boundaries with abundant defects are reduced, further reducing defects as well as nonradiative recombination centers, which is beneficial for achieving enhanced photodetection performance.^[^ ^23^, ^27^ ^]^ Then we compared the crystallization quality of perovskite film of the control and TAH samples by the X‐ray diffraction (XRD) patterns as presented in Figure 2c. It is apparent that all the perovskite films exhibited similar crystallographic orientation and showed perovskite characteristic peaks at about 14° and 28° corresponding to the (110) and (220) planes of mixed Sn–Pb perovskite, which indicate that TAH is not incorporated into the perovskite crystal structure.^[^ ^22^ ^]^ In comparison with the control film, the higher intensity values of the (110) characteristic peak for the TAH samples indicate enhanced perovskite crystallinity which is in good agreement with the SEM and AFM results. Notably, in comparison with control film, TAH sample had no peak of PbI_2_/SnI_2_ at 12.67° (Figure 2c), which clearly indicates that TAH can effectively reduce the PbI_2_/SnI_2_, further decreasing the recombination rate at the grain boundaries and surface of TAH sample, thereby improving the performance of the devices.^[^ ^29^ ^]^ These findings clearly show that TAH could effectively slowdown the crystallization process and get a well‐preserved crystal structure, which is potentially attributed to the TAH having multifunctional groups and demonstrates the simultaneous intense chemical bonding with cation (FA^+^, Pb^2+^/Sn^2+^) and anion (I^−^) in perovskite.

For Sn‐containing perovskites, the suppression of Sn^2+^ oxidation is very important as the oxidation leads to abundant defects which is harmful for the film quality and optoelectrical performance of device. To evaluate the antioxidation effect of TAH in Pb‐Sn perovskite film, XPS was carried out to analyze the chemical valence state of Sn element and the corresponding spectra are shown in Figure 2d,e. The deconvoluted Sn‐spectra in control sample showed four main peaks indicating Sn^4+^ chemical state at 487.4 and 495.8 eV and Sn^2+^ chemical state at 486.7 and 495.2 eV.^[^ ^23^, ^27^ ^]^ When TAH was introduced, the Sn^2+^ chemical state shifted to higher binding energy of 0.3 eV, which is consistent with the XPS analysis mentioned above. Additionally, the control sample exhibited 62.8% Sn^4+^ area ratio, whereas the TAH sample only exhibited 27.4% Sn^4+^ area ratio (Figure 2f), implying that TAH has effectively restrained the oxidation of Sn^2+^ to Sn^4+^ owing to the antioxidant property of hydrazine group in TAH, and TAH preferably interacts with Sn, further exhibiting effective passivation for Sn‐related defects.

### 2.3 Mechanism of Enhanced Performance Based on FPA Strategy

**Figure 3:**
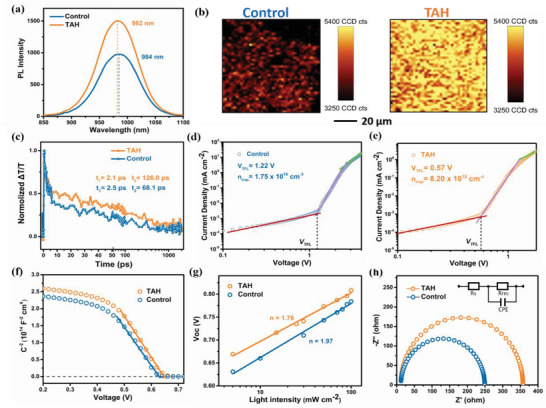
Defect passivation mechanism of functional passivating antioxidant (FPA) strategy in mixed Sn–Pb perovskite film. a) Steady‐state photoluminescence (PL) spectra of control and thiophene‐2‐carbohydrazide (TAH) films. b) Normalized PL mapping of control and TAH films. c) Transient absorption (TA) study of control and TAH films on a glass substrate. d,e) Space charge limited current (SCLC) measurements of hole‐only devices. f) Mott–Schottky (M–S) plots of the control and TAH devices. g) The measured *V*_oc_ versus light intensity plots (dots), along with the linear fits (solid lines) to the data of control and TAH devices. h) electrochemical impedance spectroscopy (EIS) of the control and TAH devices.

To clarify the underlying physical mechanism of FPA strategy in improving the photodetection performance and stability, we firstly investigated the effects of TAH on charge dynamics of perovskite. The steady‐state photoluminescence (PL), PL mapping, and femtosecond transient absorption (TA) spectroscopy of the control and TAH films deposited on the quartz were performed to analyze the carrier recombination. Due to the low TAH concentration (<2.0 mg mL^−1^), it has been found that the difference of absorption between control and TAH sample is not very obvious (Figure S5, Supporting Information). However, the TAH sample (1.2 mg mL^−1^ concentration) showed significantly enhanced PL intensity and a slight blueshift in PL peak (2 nm) compared to the control sample (**Figure** **3**a), which indicates the suppression of spontaneous nonradiative charge recombination induced by the trap states through passivating the defects. The TAH concentration was optimized to be (1.2 mg mL^−1^) (Figure S6, Supporting Information) because the lower concentration (0.5 mg mL^−1^) did not have significant effect on PL intensity, while increasing the concentration (2.0 mg mL^−1^) resulted in lower PL intensity than control sample. The effect of TAH on film uniformity was elucidated by the 2D PL mapping imaging as shown in Figure 3b. The PL mapping images confirm that TAH film shows stronger PL emission than the control one, consistent with the steady‐state PL spectra. Additionally, more uniform PL intensity distribution was found in TAH film than the control one, which is attributed to the uniform crystallization of films and the reduction of defects at both the surface and grain boundaries. To further study the effects of TAH on the charge transfer dynamics in perovskite films, femtosecond TA spectroscopy was employed. As shown in Figure 3c, a longer decay lifetime has been observed in TAH film than the control one. We further analyzed data using curves fitting by exponential function of second order. A fast lifetime *t*_1_ and a slow lifetime *t*_2_ can be resolved in Figure S7 (Supporting Information). The fast lifetime is related to the hot carriers cooling process and the slow lifetime is related to the radiative process of carriers. The fast lifetime *t*_1_ showed no significant difference in both the control and TAH films, while the slow lifetime *t*_2_ in TAH film is almost double than the control one, which implies that less carriers in TAH films proceed to nonradiative recombination associated with trap states in the band gap.^[^ ^26^, ^30^ ^]^

In addition, TAH can efficiently reduce the defects in surface and grain boundaries of films. To elucidate the defects passivation of TAH in the perovskite films, the trap density (*N*_t_) of hole‐only devices was investigated using the space charge limited current (SCLC) method. While hole‐only device was fabricated with the structure of ITO/poly(3,4‐ethylenedioxythiophene):poly(styrenesulfonate) (PEDOT:PSS)/perovskite/Spiro/Au. As shown in Figure 3d,e, the *V*_TFL_ and *N*_t_ of devices are 1.22 V and 1.38 × 10^16^ cm^3^ in control device while they reduced to 0.57 V and 6.73 × 10^15^ cm^3^ in TAH device, which clearly depicts the defects passivation effect of TAH in perovskite films. Moreover, the Mott–Schottky (M–S) analysis by capacitance–voltage (*C*–*V*) measurements was used to directly compare the built‐in potential (*V*_bi_) of PDs. As shown in Figure 3f, the PD with TAH shows a higher *V*_bi_ of 0.645 V than a 0.626 V of control device, which improves the internal driving force of carrier transfer to enhance the extraction of carriers, further improving the photodetection performance.^[^ ^27^ ^]^ Moreover, the slope of the curve is decreased in TAH devices as compared to the control device, indicating the reduction of p‐type doping in TAH film owing to the anti‐oxidation function of TAH, which is consistent with the low dark current in TAH devices.^[^ ^27^ ^]^ Further, the *J*_sc_ and *V*_oc_ as the function of illumination intensity were measured. In the Figure S8 (Supporting Information), the values of the exponential factor (*α*) are 0.994 and 0.967 for TAH and control device, respectively, which imply they both exhibit similar bimolecular recombination. In the Figure 3g, the slop of *V*_oc_ as a function of illumination intensity is 1.76 kT q^−1^ in TAH device, which is lower than the 1.97 kT q^−1^ in control device, where *k* is the Boltzmann constant and *T* is temperature. Generally, the *n* value in nkT/q indicates the trap‐assisted nonradiative recombination in devices, and the smaller *n* value indicates the less traps, owing to the fact that traps have been effectively suppressed by TAH passivation.^[^ ^23^ ^]^ Furthermore, the electrochemical impedance spectroscopy (EIS) of the corresponding devices was used to analyze how TAH impacts the charge dynamics. The Nyquist plots of devices indicate the dielectric polarization in the bulk perovskite, which can be fitted using the equivalent circuit such as the insert in Figure 3h. The series and recombination resistance (*R*_s_ and *R*_rec_) can be extracted at high and low frequencies, respectively, and the values are shown in Table S2 (Supporting Information). The TAH device showed the higher *R*_rec_ and lower *R*_s_ than the control one, which imply that the TAH can effectively reduce the bulk recombination in PDs.^[^ ^26^ ^]^

### 2.4 Effect of FPA Strategy on the Performance and Stability of Self‐Powered MSP PPDs

**Figure 4:**
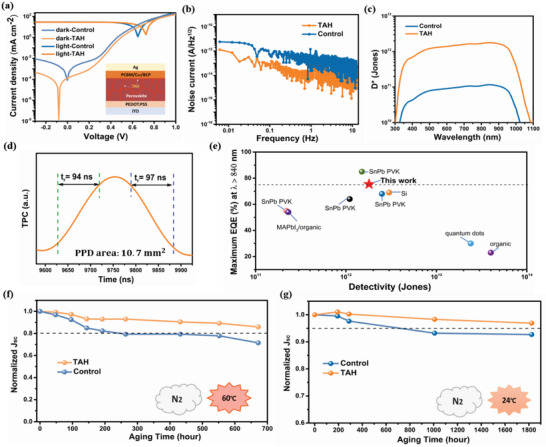
Effect of functional passivating antioxidant (FPA) strategy on performance and stability of mixed Sn–Pb perovskite photodetectors (PPDs). a) Dark and light *J*–*V* curves of the mixed tin‐lead (MSP) PPDs. The inset shows the inverted architecture of the fabricated devices. b) Noise current of the MSP PPDs at 0 V. c) Detectivity of the MSP PPDs at 0 V. d) Response time of the MSP PPDs at 0 V. e) Plot of the maximum external quantum efficiency (EQE) for wavelengths >840 nm versus the detectivity at 0 V. f) Heat stability and g) shelf stability of the mixed Sn–Pb PPDs.

To understand the effect of the TAH on photodetection performance, a vertical self‐power PD was fabricated with a structure of glass/tin oxide (ITO)/PEDOT:PSS/FA_0.7_MA_0.3_Pb_0.5_Sn_0.5_I_3_/PCBM/fullerene (C60)/bahocuproine (BCP)/silver (Ag) using perovskite and TAH‐containing perovskite, respectively (**Figure** **4**a inset). EQE was used to evaluate the optoelectronic conversion capability of PD, which is defined as the ratio between the number of photogenerated carriers and the incident photons under incident light on the specific wavelength.^[^ ^10^ ^]^ For TAH‐containing device, a higher EQE of over 75% was obtained in the NIR region (800–840 nm) than the control device under zero bias (Figure S9, Supporting Information). To be precise, the maximum EQE values of TAH‐containing devices and control devices in NIR region (>800 nm) are 75.6% and 74.7%, respectively, at 820 nm wavelength of light. The responsivity (*R*) is another important parameter to evaluate the light detecting ability of PDs,^[^ ^21^ ^]^ which is defined as the photocurrent through the detector per active area per unit power of light and can be expressed from EQE by

(1) $$R_{\lambda }=\frac{\mathrm{EQE}\lambda q}{hc}$$

where *λ* denotes the wavelength of incident light, *q* is the absolute value of the electronic charge, *h* is the Planck constant, and *c* is the velocity of light in a vacuum. Figure S9 (Supporting Information) shows that the TAH‐containing PDs reached to a peak R of 0.51 A W^−1^ at an NIR light with 840 nm wavelength and 0.40 A W^−1^ at 940 nm NIR light without external bias, respectively, which are comparable to highest reported values for similar perovskite composition and traditional Si PDs.^[^ ^8^, ^21^ ^]^ Detectivity (*D* ^*^) is a parameter to evaluate the sensitivity and anti‐noise ability, which is another key parameter of high‐performance PD, which can be given by

(2) $$D^{*}=\frac{\sqrt{AB}}{NEP}$$

The noise equivalent power (NEP) implies the lowest light intensity that PDs can distinguish from noise and the calculation formula is as follows

(3) $$\mathrm{NEP}=\frac{i_{n}}{R}$$

where *i_n_* denotes the total noise current, *R* represents the responsivity, *A* stands for active area of devices, and *B* represents the bandwidth. A significant challenge in achieving high D* is lowering the dark current density (*J*_D_) and noise current (*i*_n_).^[^ ^8^ ^]^ Therefore, an effective suppression of dark current can significantly contribute to achieving PDs with high D*. Figure 4a and Figure S10 (Supporting Information) show the current–voltage curve of control and TAH‐containing PDs under dark. Notably, it is clearly seen that TAH‐containing PPDs shows significantly reduced dark current. The lowest dark current density of the TAH‐containing PPDs is 1.5 × 10^−8^ mA cm^−2^ at – 0.07 V bias, which is almost three orders of magnitude lower than that of control PPDs (7.9 × 10^−5^ mA cm^−2^). This result further implies that defects passivation toward perovskite by TAH can efficiently suppress dark current. While, enhanced *V*_oc_ manifest that the defects were passivated and nonradiative recombination was reduced.^[^ ^27^ ^]^ The photocurrent *J*_P_ is 28.44 mA cm^−2^ in TAH‐containing PPDs under 1 sun at 0 V bias, corresponding to a *J*_P_/*J*_D_ ratio of around 10^9^. It is known that noise refers to the random fluctuations in the average value of the signal, and it has three types namely shot noise, thermal noise, and *1/f* noise.^[^ ^10^, ^31^ ^]^ The *1/f* noise is related to frequency, while the other two are independent of frequency.^[^ ^31^ ^]^ The total noise current of mixed Sn–Pb PPDs was measured under zero bias from the fast Fourier transform of dark current as a function of time, as shown in Figure 4b. Obviously, the total noise current of both PPDs is dominated by 1/*f* noise in the low frequency range, that is mainly derived from the local electronic state fluctuations caused by disorders or defects.^[^ ^31^ ^]^ For control PPDs, the noise current is 1.42 × 10^−12^ A Hz^−1/2^ at 1 Hz, while the TAH‐containing PPDs significantly suppressed the *1/f* noise and showed the value of 9.36 × 10^−14^ A Hz^−1/2^ at 1 Hz, which indicates that the effective defects passivation can enable low total noise currents. Combining the R and noise current, the D* curves with wavelengths are obtained in Figure 4c. The specific detectivity D* of the TAH containing PPDs was calculated to be over 1.2 × 10^12^ in the broad spectral range (500–950 nm), with a maximum of 1.8 × 10^12^ Jones in the NIR region (at 840 nm), which is around 10‐fold higher than that of control PPDs (1.2 × 10^11^ Jones). The D* value of the TAH containing PPDs is comparable to the best NIR solution processed PDs using organics, quantum dots as shown in Figure 4e and Table S1 (Supporting Information). Notably, the D* value of the TAH containing PPDs is also very much close to the one of commercial Si based PDs (3 × 10^12^ Jones),^[^ ^21^ ^]^ which implies its potential applications in weak NIR light detection.

The response speed represents the ability of the PDs to track fast‐changing light signals. The rise time is defined as the time that is required to increase from 10% to 90% of maximum photocurrent, and the decay time is the time that is required to decrease from 90% to 10% of maximum photocurrent. The response speed of PDs is also defined as *f*_3dB_ in optical fiber communication which is mainly limited by the resistance–capacitance (RC) time constant and the transit time (*t*_tr_), expressed by

(4) $$f_{3dB}^{-2}={\left(\frac{3.5}{2\pi t_{\mathrm{tr}}}\right)}^{-2}+{\left(\frac{1}{2\pi RC}\right)}^{-2}$$

The RC time constant is induced by the resistance and parasitic capacitance, which inversely corelates with the device area, which means that relatively smaller device area can induce faster response, due to the parasitic capacitance limitation of devices.^[^ ^7^ ^]^ However, in our case, despite of the bigger device area, our response time was significantly faster. Herein, the transient photocurrent (TPC) was measured as the response speed with a nanosecond Sinc function pulsed laser (450 nm). The pulse signal was responded by TAH‐containing PPDs from the nanosecond pulsed laser under zero bias and then the TPC curve was recorded by an oscilloscope (Figure 4d). The photocurrent rapidly increases and decays in response to the light pulses, which exhibited a tens of nanosecond rise/fall time of 94 and 97 ns in 10.7 mm^2^ TAH‐containing PPDs. This response speed in such a large area PDs is comparable to the best reported NIR PPDs with small devices area, which proves the significance of this work (Table S1, Supporting Information).

The stability of the PDs is very important in practical applications, especially the operating, heat, and shelf stability. Firstly, the heat stability of unencapsulated mixed Sn–Pb PPDs was carried out by continuously heating devices on 60 °C under N_2_ glove box, as shown in Figure 4f. The control devices showed 71.4% of initial photoresponse after 672 h of continuous heating, while the TAH devices still retained 85.9% of initial photoresponse. The improved thermal stability can be attributed to the hydrogen bonds between FA^+^ and TAH, due to the volatile organic cations in perovskite are the main cause of instability under heat pressure,^[^ ^32^ ^]^ which has been successfully suppressed by the hydrogen bonds between FA^+^ and TAH. In addition, the shelf stability of devices in N_2_ glove box is also recorded, as shown in Figure 4g. The TAH devices have shown a remarkable storage stability retaining 96.9% of initial photoresponse after 1824 h storage. Whereas, the control devices decayed to 92.6% of initial photoresponse after 1824 h storage. We also evaluated the effect of TAH on the storage stability in air (the humidity level of 30%, and the temperature of 25 °C). As shown in Figure S11 (Supporting Information), the TAH PPDs exhibited better air stability than control devices, which not only stemmed from the hydrophobic organic group of TAH that have increased the ability to isolate water from the oxygen of perovskite (Figure S12, Supporting Information) but also the anti‐oxidation of TAH (Figure 2f). These results imply that the TAH PPDs have long‐term stability with no extra encapsulation.

### 2.5 Self‐Powered Flexible Mixed Sn–Pb (FMSP) PPDs with High Performance and Stability

**Figure 5:**
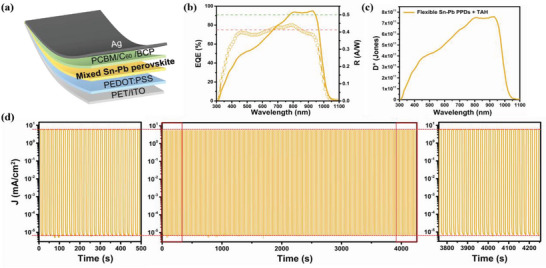
Performance and stability of flexible mixed Sn–Pb (FMSP) perovskite photodetectors (PPDs). a) Schematic diagram of the FMSP PPDs. b) The external quantum efficiency (EQE) and responsivity of the FMSP PPDs at 0 V. c) Detectivity of the FMSP PPDs at 0 V. d) Continuous track of the photo and dark current of the FMSP PPDs under 0 V bias during a period of over 4000 s.

For the first time to the best of our knowledge, the flexible mixed Sn–Pb (FMSP) PPDs were fabricated owing to excellent photodetection of rigid MSP PPDs with TAH. A vertical self‐powered FMSP PPDs with TAH was fabricated by replacing glass substrate with polyethylene terephthalate (PET) substrate (**Figure** **5**a). It's worth noting that the FMSP PPDs exhibited the maximum EQE (80.1%) at 790 nm and over 70% EQE at the NIR range from 800 to 920 nm. To be precise, the EQE of the FMSP PPDs is 75% in 840 nm and 70% at 920 nm wavelength of light, respectively (Figure 5b). This corresponds to two peaks of responsivity at 810 and 930 nm of 0.52 A W^−1^, which is comparable with the highest reported value (0.6 A W^−1^ at 890 nm) for PIN perovskite NIR PDs with rigid substrate and is the highest value in flexible PIN PPDs at NIR range (>840 nm).^[^ ^21^, ^33^ ^]^ The noise spectral density of the FMSP PPDs was calculated (2.3 × 10^−13^ A Hz^−1/2^ at 1 Hz) by the dark currented measured at 0 V, which is 1/f dependence in the low frequency region (Figure S13, Supporting Information). Combining the responsivity and noise current, the detectivity of the FMSP PPDs was calculated to be over 7.4 × 10^11^ Jones, with a maximum of 7.5 × 10^11^ Jones in 810 and 930 nm NIR range (Figure 5c). The on‐off stability is an important parameter for PDs, which shows the capacity of device to stably operate. The unencapsulated FMSP PPDs were tested for on‐off stability measurement under N_2_ atmosphere (Figure 5d). The *J*_p_ and *J*_d_ of the FMSP PPDs showed no any attenuation during working time of 4000 s, with the large stable on‐off ratio of 10^6^. This indicates that the FMSP PPDs showed profound light stability, which can be ascribed to less residual PbI_2_ in TAH films.^[^ ^34^ ^]^ Some intensive studies have demonstrated that residual PbI_2_ in perovskite is detrimental to the light stability of perovskite‐based devices. PbI_2_ can decompose into metallic Pb under the stimuli of light. Importantly, metallic Pb as a primary deep defect state can deteriorate the performance and long‐term stability of perovskite‐based devices.^[^ ^35^ ^]^ In addition, the mechanical stability of the FMSP PPDs was investigated. The encapsulated FMSP PPDs were bent with a curvature radius of 3 mm for cycle index in air (Figure S14, Supporting Information). The devices after 800 bend cycles still kept 90% of initial photocurrent, which shows the excellent flexibility of FMSP PPDs.

### 2.6 Wearable Mixed Sn–Pb PPDs for Remote Health Monitoring

**Figure 6:**
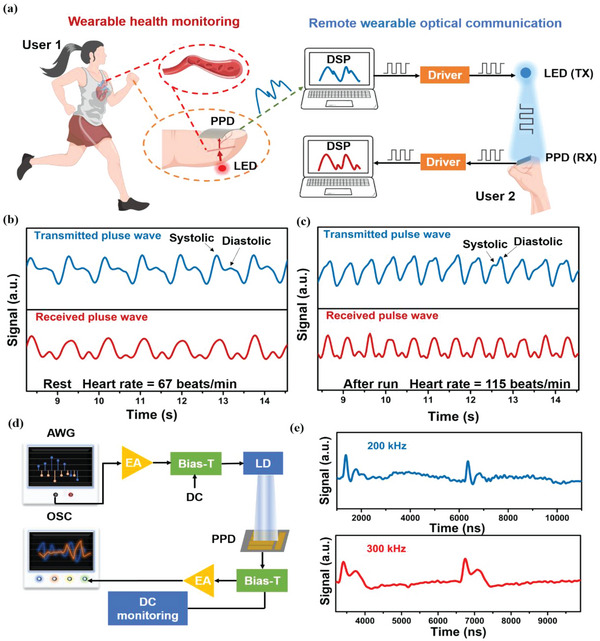
Demonstration of the self‐powered flexible mixed Sn–Pb (FMSP) perovskite photodetectors (PPDs) for wearable biomedicine and optical communications integration. a) Schematic illustration of the application of FMSP PPD in wearable remote health monitoring. (TX: transmitter RX: receiver) b,c) the pulse signal measured from the FMSP PPD (transmitted pulse signal) and the received pulse signal by optical communication at rest and after‐run conditions. d) The setups of the high frequency optical communication. e) Transient light response of FMSP PPD under light modulation frequency of 200 and 300 kHz without extra bias.

Finally, we developed a system to achieve wearable remote health monitoring via the self‐powered FMSP PPDs as the wearable receivers on two different users. This system includes wearable health monitoring and remote wearable optical communication modules (**Figure** **6**a). In wearable health monitoring module, we carried out a simple photoplethysmography (PPG) experiment using the FMSP PPD to monitor the pulse and determine the heart rate. The working principle of this PPG test is that the light (850 nm) emitted from LEDs is partially absorbed, reflected, and scattered by human tissues which can be detected by the FMSP PPD worn on the human body (user 1). Hence, the change of blood volume upon each cardiac cycle can be shown to monitor the pulse wave and estimate the heart rate. In remote wearable optical communication module, the monitored pulse waves are modulated to the on/off status of blue LED, which is the transmitter. The modulated light is then received by the other FMSP PPD worn on the user 2 and then transferred to the computer. The photograph and videos of those two parts are shown in Figure S15 and Video S1 and Video S2 (Supporting Information). The pulsatile signals of user 1 (one of the authors) were remotely monitored by user 2 (one of the authors) at rest and after‐run conditions, respectively (Figure 6b,c). It is noteworthy that data were collected under the informed consent of these authors, which is mentioned in the Experimental Section also. In both conditions, the transmitted pulse waves showed the typical systolic and diastolic peaks in a PPG profile and the received pulse wave kept the synchronization and similar profile with transmitted pulse waves, which indicates high sensitivity and detectivity of the FMSP PPDs. The heart rate can be calculated to be 67 and 115 beats min^−1^ in transmitted and received pulse waves at rest and after‐run conditions, respectively. In addition, the high frequency optical communication test was also carried out to demonstrate the fast response speed of FMSP PPDs. Figure 6d shows the setups of the high frequency optical communication test. Figure 6e shows the photocurrent signal as a function of time for PPD under optical light at 0 bias modulated at 200 and 300 kHz Sinc signal. We can observe that pulse broadening occurred due to the bandwidth limitation effect. The pulse broadening became severe as the transmitting rate increased. When the light modulation is at 200 kHz, the PPDs can follow the decay of Sinc peak, while the PPD cannot fully follow the decay of Sinc peak at 300 kHz light modulation due to the bandwidth limitation effect, which is consistent with cutoff frequency (*f_−_*_3dB_) of 313 kHz (Figure S16, Supporting Information). These results show that the FMSP PPDs can achieve audio transmission in optical communication, which shows great potential of the FMSP PPDs in wearable remote health monitoring systems between the two distinguished humans.

## 3 Conclusion

In conclusion, we, for the first time, demonstrated a novel FPA strategy to improve crystallization, restrain Sn^2+^ oxidization, and passivate defects simultaneously in MSP perovskite film by multiple interactions between TAH molecules and cations/anions in MSP perovskite. Consequently, the self‐powered rigid MSP PPD simultaneously achieved high EQE (75.4%), responsivity (0.51 W A^−1^), and detectivity (1.8 × 10^12^ Jones) in the NIR region. The device also displayed an ultrafast response time of 94 ns/97 ns. Moreover, the optimum device showed significantly improved overall stability under thermal, air, and N_2_ atmosphere. Notably, for the very first time, a self‐powered flexible MSP PPD with TAH molecules exhibited high EQE (75% at 840 nm), high responsivity (0.52 W A^−1^ at 930 nm), high detectivity (7.5 × 10^11^ Jones in 930 nm), and on‐off stability. Finally, the self‐powered flexible MSP PPDs were used for the first time in remote wearable health monitoring applications to achieve interactive‐information between different humans. The self‐powered flexible MSP PPDs meet the requirement in term of mechanical flexibility, broadband detection, and performance (EQE, detectivity, and response speed), that make them competitive in wearable health monitoring applications with low power consumption.

## 4 Experimental Section

### Materials

Tin iodide (SnI_2_, 99.999%) was purchased from Alfa Aesar. DMSO (99.8%), *N*,*N*‐dimethylformamide (DMF, 99.8%), tin fluoride (SnF_2_, 99%), TAH (98%), chlorobenzene (CB anhydrous, 99.8%), and tin powders (<150 µm, 99.5%) were purchased from Sigma–Aldrich. Methylammonium iodide (MAI), FAI, and lead iodide (PbI_2_) were purchased from GreatCell Solar. Fullerene (C60) and bathocuproine (BCP) were obtained from Xi'an Polymer Light Technology Co. Ltd., China. PEDOT:PSS aqueous solution (Al 4083) was acquired from Heraeus Clevios. All the chemical reagents were used as received without any further purification.

### Preparation of Mixed Tin–Lead Perovskite Precursor Solution

The perovskite solution having the formula of FA_0.7_MA_0.3_Sn_0.5_Pb_0.5_I_3_ was employed in this work. The FAI (1.12 m), MAI (0.48 m), SnI_2_ (0.8 m), PbI_2_ (0.8 m), and SnF_2_ (0.08 m) were dissolved into the mixed solvent of DMSO and DMF (volume ratio of 1:4) to prepare the 1.6 m perovskite solution. Varied concentrations (0, 0.5, 0.8, 1.2, and 1.6 mg mL^−1^) of TAH were fully dissolved into the perovskite precursor solution to prepare the “Control” and “TAH” solutions, respectively. The precursor solutions were stirred for 2 h at room temperature. Further, tin (Sn) powder was added into the precursor solutions to restrain Sn^2+^ oxidation. All solutions were filtered by a 0.22 µm membrane before fabricating films.

### Fabrication of Mixed Sn–Pb PPDs

The pre‐patterned ITO glass substrates were sequentially cleaned with deionized (DI) water, acetone, and ethanol by ultrasonication for 20 min followed by N_2_ blow‐dry. The substrates were further treated with UV–O_3_ for 15 min. The PEDOT:PSS solution was deposited onto the ITO substrate by spin‐coating the solution at ITO at 4000 rpm for 30 s and annealed on a hotplate for 60 min in ambient air. Subsequently, two‐steps spin‐coating method was employed to deposit perovskite precursors at 1000 r.p.m. for 10s; 4000 r.p.m. for 40 s. During the second step, 210 µL of CB was dripped onto the spinning substrates. The substrates were then annealed at 100 °C for 10 min and after cooling down to room temperature, C60 (20 nm)/BCP (3 nm)/Ag (100 nm) were deposited on top of the perovskite by thermal evaporation (Kurt).

### Fabrication of Flexible Mixed Sn–Pb PPDs

The prepatterned ITO PET substrates were sequentially cleaned with acetone, and ethanol by ultrasonication for 20 min followed by N_2_ blow‐dry. The PDMS was spin‐coated on the glass substrate, and the PET/ITO was conglutinated on the PDMS/glass substrate. The substrates were further treated with O_2_ plasma for 5 min. Further, same steps were followed which are described for mixed Sn–Pb PPDs.

### Characterization of Mixed Sn–Pb PPDs

Current density–voltage (*J*–*V*) characteristics of mixed Sn–Pb PPDs were obtained between −0.3 to 1.0 V with voltage steps of 20 mV by using a Keithley 2600 source without any preconditioning in the N_2_ glove box. The devices were illuminated using white light source xenon lamp (Oriel solar simulator, 94023A) with AM 1.5 G condition at an intensity of 100 mW cm^−2^. The EQE measurements were carried in the 300–1100 nm region by using QE‐R system. Monochromatic light intensity was calibrated by a NIST‐traceable Si detector before testing. For EQE measurements, the PPDs were kept in ambient condition (RH: 30%). Noise measurements were performed from the fast Fourier transform of dark current as a function of time under zero bias in dark conditions.

### Stability Tests of Mixed Sn–Pb PPDs

The long‐term shelf and heat stability of PPDs (without encapsulation) in N_2_ glove box was investigated by repeating the *J*–*V* measurements within different time intervals. The atmospheric stability of PPDs (without encapsulation) was tested by repeating *J*–*V* characterizations after storing in ambient air (RH: 30%; environmental temperature: 25 °C) for different time intervals.

### Space–Charge Limited Current

The hole‐only devices were fabricated to acquire the density of hole traps with the architectures: ITO/PEDOT‐PSS/perovskite/Spiro‐OMeTAD/Au, where the Spiro‐OMeTAD was doped with tBP, Co (III) TFSI, and Li‐TFSI. The *J*–*V* curves were obtained using a Keithley 2600 Source Meter in a glovebox. The trap density *N*_trap_ was determined by the equation

(5) $$n_{\mathrm{trap}}=\frac{2\varepsilon_{0}\varepsilon V_{\mathrm{TFL}}}{ed^{2}}$$

where *V*_TFL_ is the trap‐filled limit voltage, *d* is the thickness of the perovskite film (650 nm), *ɛ* is the relative dielectric constant of perovskite (54.87),^[^ ^27^ ^]^ and *ɛ_0_* is the vacuum permittivity.

### Transient Absorption Spectroscopy

TA spectroscopy measurement was carried out by using a Ti:Sapphire amplifier laser system (Spectra‐Physics Lasers, 1 kHz, 100 fs). The pump pulses (570 nm) were generated from a TOPAS‐Prime optical parametric amplifier (Light Conversion). The probe light was generated by focusing an 800 nm beam onto a sapphire plate and then decomposed into monochromatic wavelength (950 nm) through filters. The time delay between the pump and probe light was realized by controlling a stepper motor to tune their optical path differences. The transmitted probe signal was introduced into a silicon photodiode connected with a lock‐in amplifier to record the changes in transmission intensity (Δ*T*/*T*) induced by pump light.

### Other Characterizations

SEM images were obtained using field‐emission SEM (ZEISS Sigma HD) with an accelerated electron beam at 8 kV. XRD patterns were recorded using a Rigaku MiniFlex 600 diffractometer equipped with an NaI scintillation counter and using monochromatized Cu‐K*α* radiation (*λ* = 1.54 Å). X‐ray ultraviolet photoelectron spectroscopy (UPS) was performed by PHI 5000 VersaProbe III. The absorption spectra were obtained between 300 and 1100 nm wavelength range using F20‐UV thin‐film analyzer (FILMETRICS). Steady state PL spectra were obtained using a MAL‐E 405 nm excitation laser and F20‐UV thin‐film analyzer (FILMETRICS). The micro 2D PL mapping images were captured by a Raman spectrometer (WITEC Alpha300R) under 532 nm laser excitation.

### Demonstration of the Application

To demonstrate the application of both the PPG signal detector and PD for VLC system, this work firstly used the FMSP PPDs to receive the penetrated light signals from body vessel and secondly constructed a simple VLC system where the FMSP PPDs acted as the PD. The data collected from the first step was processed offline to remove the long period fluctuations by body movement and high frequency noise in the circuit. FIR band‐pass filters were designed with cut‐off frequency at 0.6 Hz/1.5 Hz or 0.6 Hz/3 Hz, depending on the participant's status (resting or regular exercising) to keep the useful PPG signal and suppress the noise for a clearer display and further processing. It is noteworthy that data were collected under the informed consent of the participants (one of the authors). To remove as much noise as possible, the FIR filter parameter such as cut‐off frequencies were selected according to status of the participants (resting or regular exercising). Next, the signals were converted into the bivalued signals, (or an OOK, on‐off keying modulation) to enhance the anti‐noise ability, using the high voltage for signals above threshold and otherwise the low one. To simplify the communication process, all OOK signal data was sent to an Arduino UNO prior to the communication process. In the communication system, a blue LED was driven to flash when signal voltage was high and meanwhile the perovskite device generated photocurrent. The Arduino UNO was used to drive the LED as well as read the perovskite device. It was programmed to update the output voltage (5 V and 40 mA at most) at its analog output pin every 30 ms as required, and simultaneously, recorded the voltage across the perovskite device at the same frequency as transmission. The received signals were later processed with another FIR filter on PC to remove the noise from the transmitter, channel, and the receiver. Later, the DSP process sequentially used the threshold detection and rising edge to locate the heart beat and finally calculate the heart rate.

In the demonstration of Sinc pulse transmission, an optical communication system was established. The setup of the optical communication system is shown in Figure 6d. First, an arbitrary signal generator (AWG, KEYSIGHT M9502A) was used to generate the Sinc waveform. Then the signal went through an electrical amplifier (EA, ZHL‐2‐8‐S+). The amplified signal was coupled with direct current to drive the blue LD (450 nm) through a bias‐Tee (ZFBT‐4R2GW‐FT+). After free‐space transmission, the PPD was employed to receive the optical signal followed by a bias‐Tee. The DC output of bias‐Tee was used for direct current monitoring. Next, after amplified by an EA, the received signal was displayed on an oscilloscope (OSC, AGILENT MSO9404A).

## Conflict of Interest

The authors declare no conflict of interest.

## Supporting information

Supporting Information

Supplemental Video 1

Supplemental Video 2

## Data Availability

Research data are not shared.
