# Supplementary material for: Highly Efficient and Stable Self‐Powered Mixed Tin‐Lead Perovskite Photodetector Used in Remote Wearable Health Monitoring Technology
Source: Adv Sci (Weinh). 2022 Dec 9;10(5):2205879. doi: 10.1002/advs.202205879 (PMC9929128; doi:10.1002/advs.202205879)
Supplement: Supplementary file 1 — Supporting Information [file ADVS-10-2205879-s003.pdf]

ADVANCED SCIENCE
Open Access

Supporting Information

for *Adv. Sci.*, DOI 10.1002/advs.202205879

Highly Efficient and Stable Self-Powered Mixed Tin-Lead Perovskite Photodetector Used in Remote Wearable Health Monitoring Technology

Fengcai Liu, Kai Liu, Saqib Rafique, Zengyi Xu, Wenqing Niu, Xiaoguo Li, Yifan Wang, Liangliang Deng, Jiao Wang, Xiaofei Yue, Tao Li, Jun Wang, Paola Ayala, Chunxiao Cong, Yajie Qin, Anran Yu, Nan Chi* and Yiqiang Zhan**

Supporting Information

Highly Efficient and Stable Self-Powered Mixed Tin-Lead Perovskite Photodetector Used in Remote Wearable Health Monitoring Technology

Fengcai Liu[1†], Kai Liu[1†], Saqib Rafique[1], Zengyi Xu[2], Wenqing Niu[2], Xiaoguo Li[1], Yifan Wang[2], Liangliang Deng[1], Jiao Wang[1], Xiaofei Yue[1], Tao Li[3], Jun Wang[3], Paola Ayala[4] Chunxiao Cong[1],Yajie Qin[1], Anran Yu[1], Nan Chi[2*], Yiqiang Zhan[1,5*]*

Affiliations:

[1]Center for Micro Nano Systems, School of Information Science and Technology (SIST), Fudan University, 200433 Shanghai, P. R. China

[2]Key Laboratory for Information Science of Electromagnetic Waves (MoE), Department of Communication Science and Engineering, Fudan University, Shanghai 200433, China

[3]Key Laboratory of Micro and Nano Photonic Structures (MOE), and Shanghai Ultra-precision Optical Manufacturing Engineering Research Center, Department of Optical Science and Engineering, Fudan University, Shanghai, 200433, China

[4]Faculty of Physics, University of Vienna, 1090 Vienna, Austria

[5]Shanghai Frontier Base of Intelligent Optoelectronics and Perception, Institute of Optoelectronics, Fudan University, 2005 Songhu Road, Shanghai, 200438, P. R. China

***Corresponding Author.**

Email: yqzhan@fudan.edu.cn; nanchi@fudan.edu.cn; aryu@fudan.edu.cn

[†]These authors contributed equally to this work.

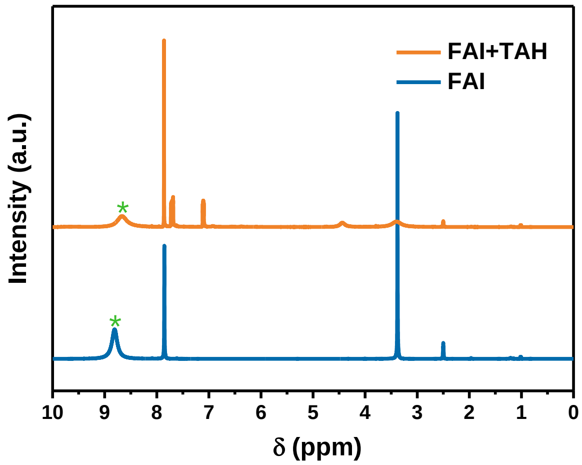

Figure S1. The full regions of ^{1}H nuclear magnetic resonance (^{1}H NMR).

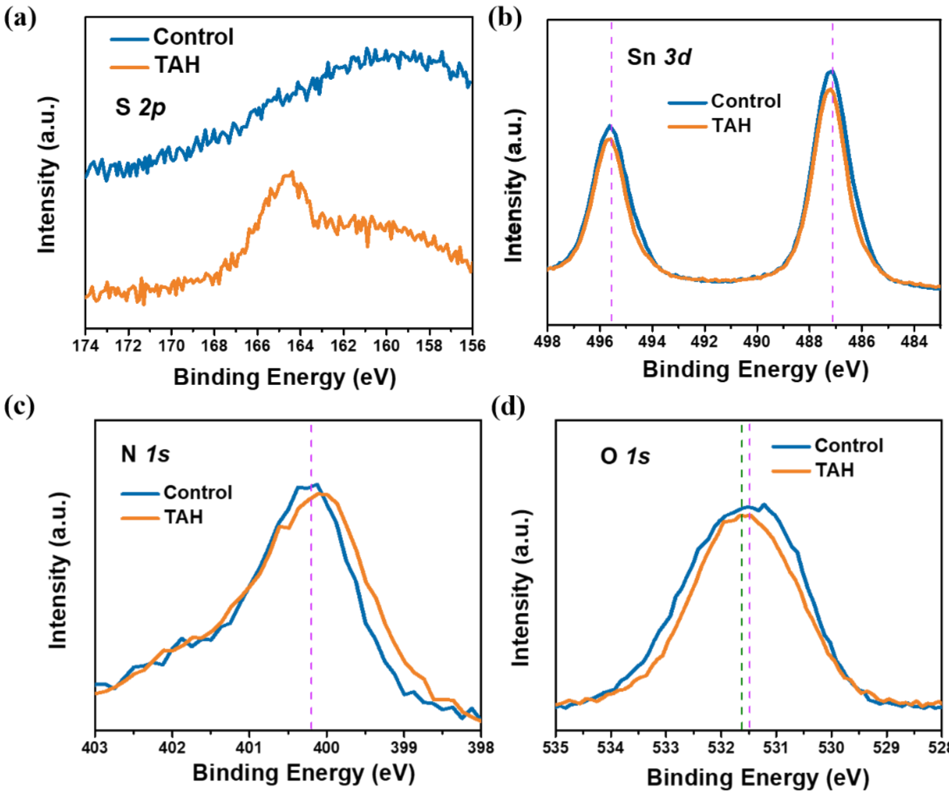

Figure S2. a, **b**, **c**, and **d**, The XPS spectra of S 2p, Sn 3d, N 1s and O 1s orbit in control and TAH films.

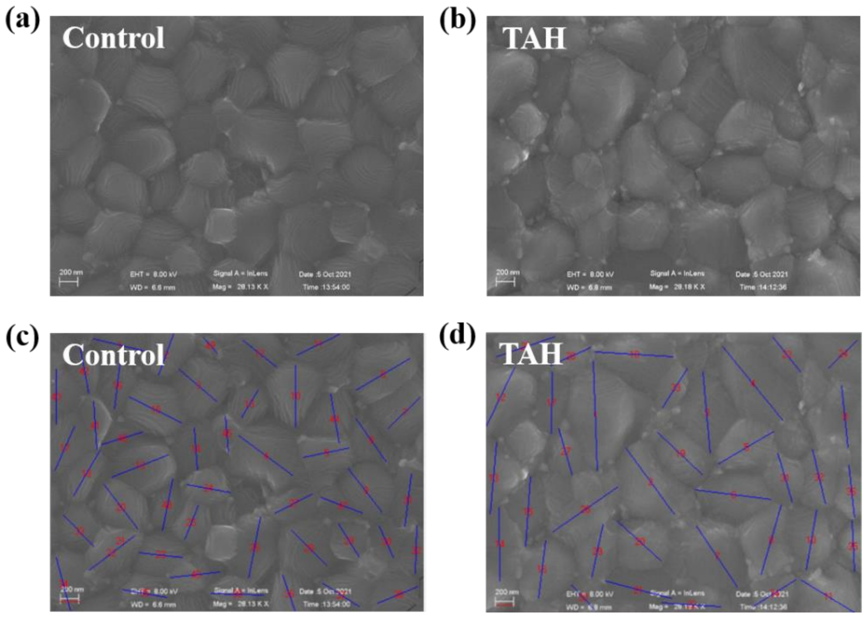

Figure S3. **(a, b)** Top-view SEM images and **(c, d)** grains size analysis of control and TAH perovskite films.

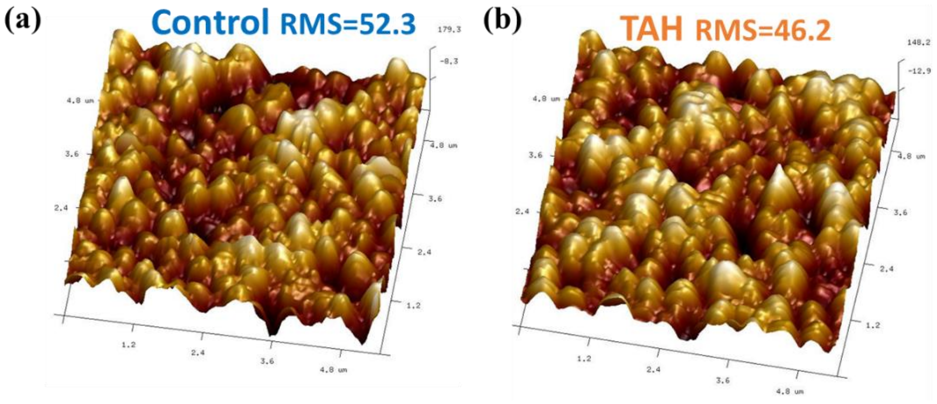

Figure S4. AFM images of control and TAH films.

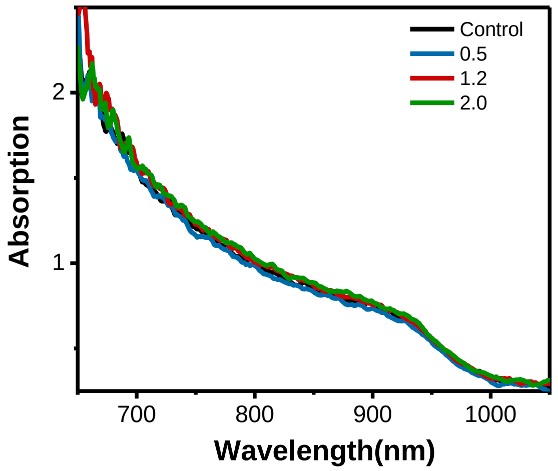

Figure S5. The absorption of the mixed Sn-Pb perovskite films with different TAH content.

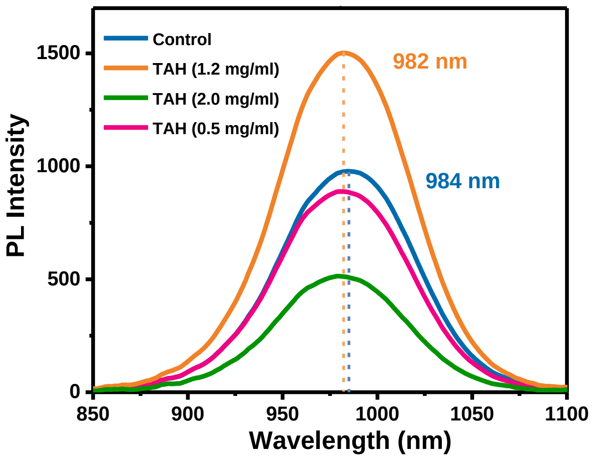

Figure S6. The PL of the mixed Sn-Pb perovskite films with different TAH content.

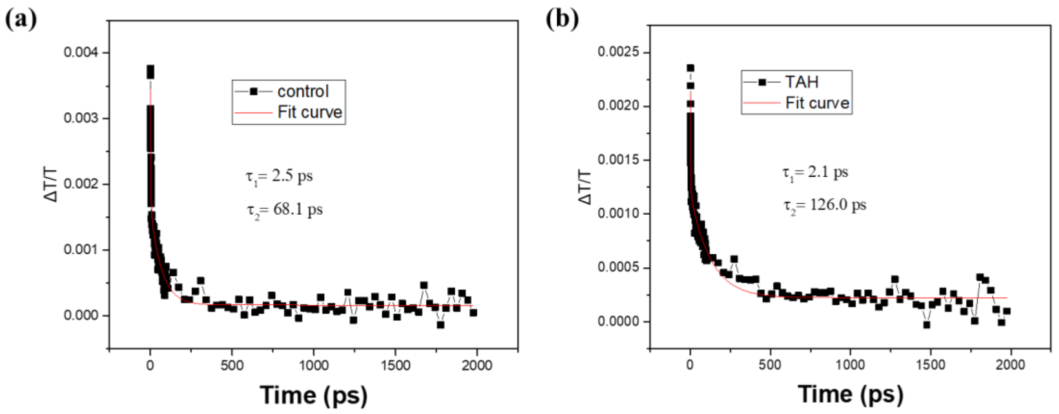

Figure S7. The Transient absorption and fit curves of control and TAH films

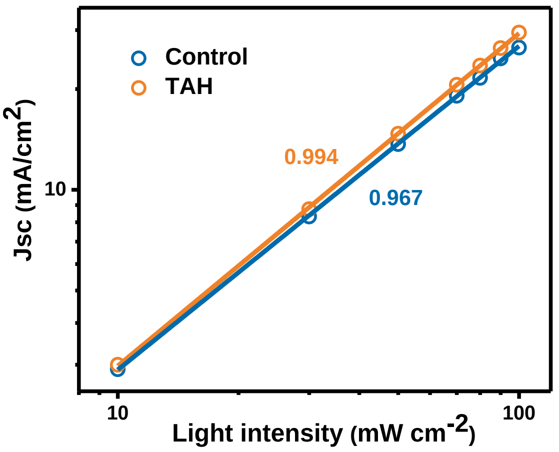

Figure S8. Measure J_{sc} plotted against light intensity (dots), together with linear fits (solid lines) to the data of control and TAH devices.

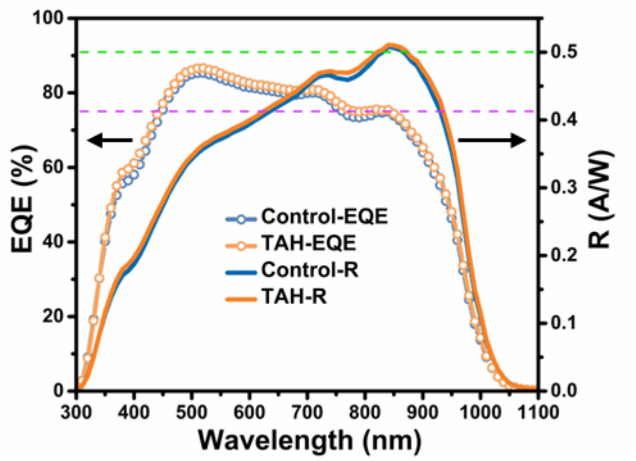

Figure S9. the EQE and responsivity of the MSPP PDs.

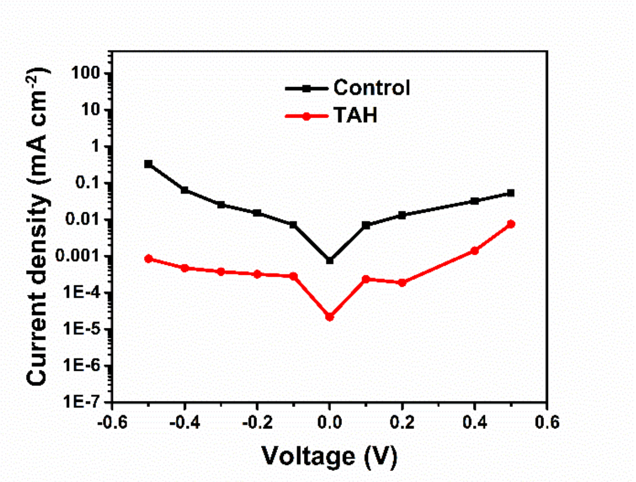

Figure S10. The single point test to test the dark current of control and TAH-containing PPDs.

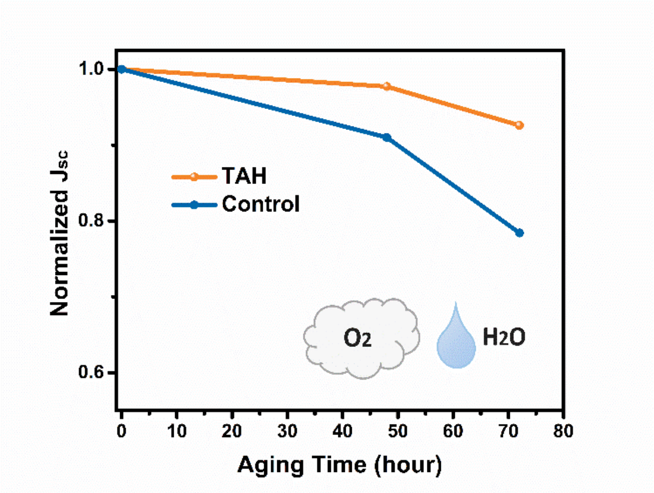

Figure S11. Air stability of the control and TAH PPDs.

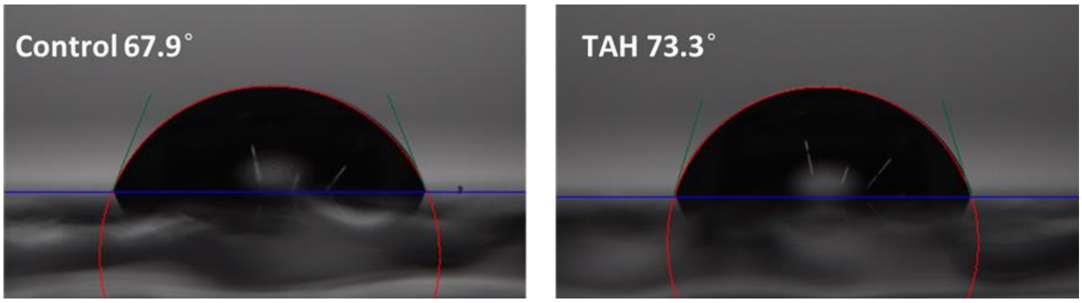

Figure S12. Water contact Angle test of of control and TAH films.

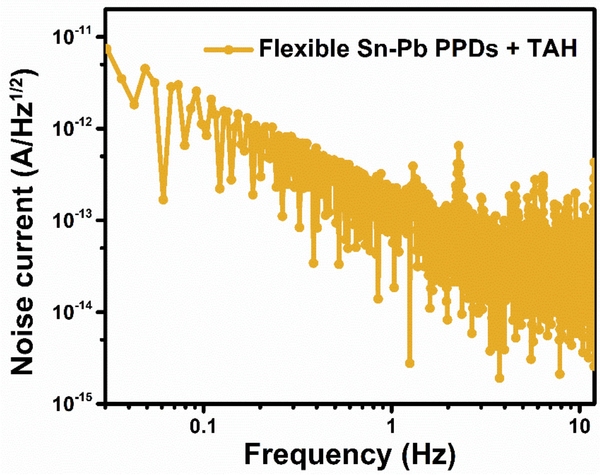

Figure S13. The noise spectral density of the FMSP PPDs.

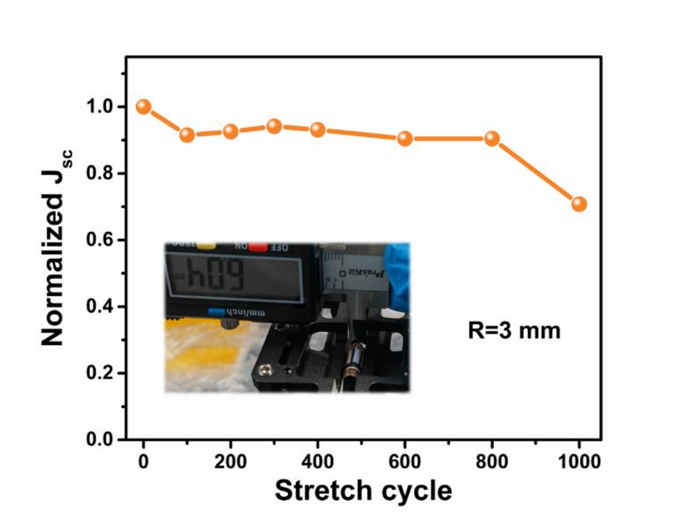

Figure S14. Normalized J_{sc} of the FMSP PPDs as a function of stretch cycle with 3 mm.

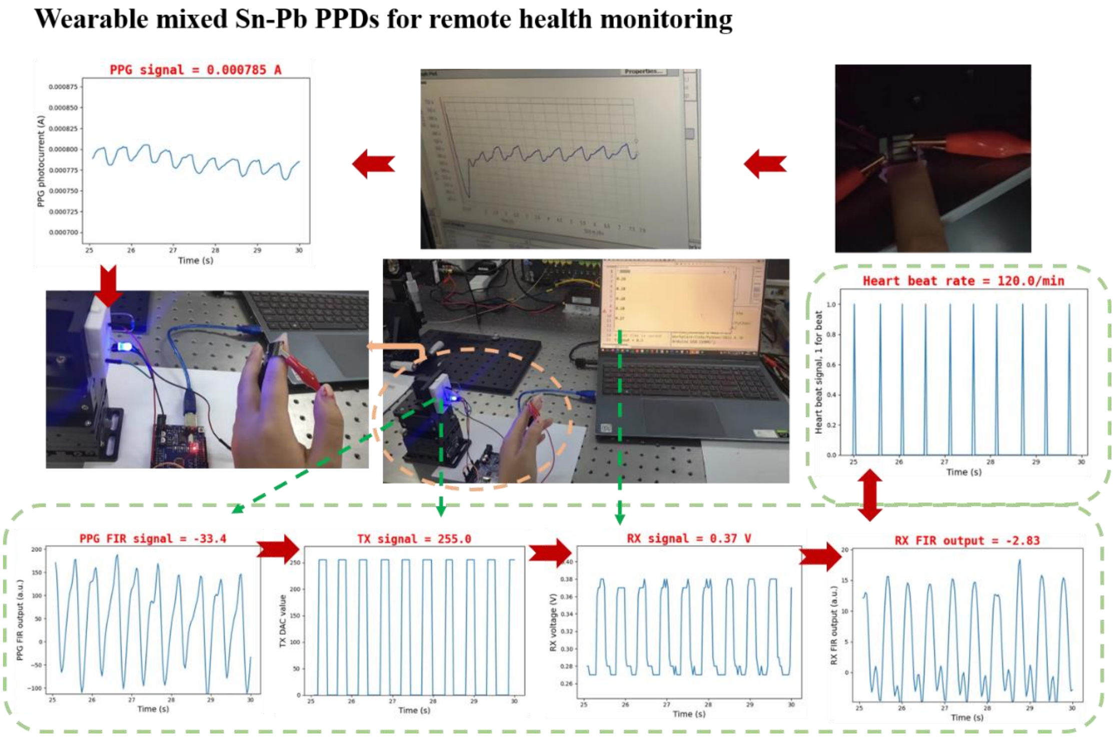

Figure S15. The photograph and monitored pulse data of wearable biomedicine and optical communications integration (wearable remote health monitoring).

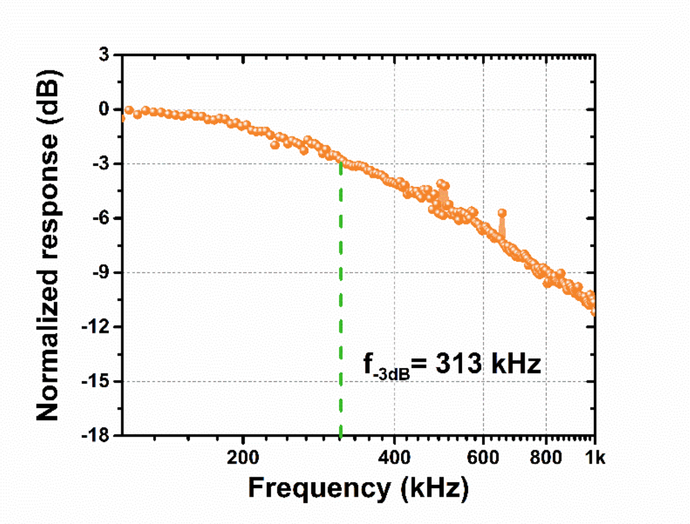

Figure S16. The bandwidth of the FMSP PPDs.

Table S1. Comparison of the solution-processed and Si photodetectors

Devices	EQE (%)(>840nm)	J_D(A/cm^2) @ 0 V	D (Jones)	Response time T_{fall} (ns)	Devices area (mm^2)	Ref.
PEDOT:PSS/ $FA_{0.6}MA_{0.4}Sn_{0.6}Pb_{0.4}I_3$/ C_{60}	64	8×10^{-9}	1.1×10^{12}*	9100	3.8	[1]
PTAA/ $Cs_{0.05}FA_{0.5}MA_{0.4}5Sn_{0.5}Pb_{0.5}I_3$/TBA-Azo/ C_{60}	55	1×10^{-8}	2.2×10^{11}*	42.9	-	[2]
PTAA:poly-TPD/ $FA_{0.66}MA_{0.34}Sn_{0.5}Pb_{0.5}I_3$/ C_{60}	68	5×10^{-12}	2.5×10^{12}*	740	1	[3]
NiO_x/ $FA_{0.7}MA_{0.3}Sn_{0.5}Pb_{0.5}I_3$/ C_{60}	85	4.5×10^{-9}	1.5×10^{12}*	150	2	[4]
PTAA/ $MAPbI_3$/ F8IC:PTB7-Th/ C_{60}	54	1×10^{-9}	2.3×10^{11}*	5.6	0.1	[5]
MoO_3/ PBTTT:PCBM/ PEIE	23	4×10^{-11}	>10^{13}†	230	0.5	[6]
MoO_x/ PbS-QD/ TiO_2	30	10^{-12}	2.4×10^{13}*	292	-	[7]
Si	69	7.9×10^{-8}	3.0×10^{12}*	0.3	0.13	[4]
**PEDOT:PSS/ $FA_{0.7}MA_{0.3}Sn_{0.5}Pb_{0.5}I_3$ +TAH/ PCBM/ C60**	**75.4**	$\mathbf{1.2 \times 10^{-10}}$	$\mathbf{1.8 \times 10^{12}}$*	**97**	**10.7**	**This work**

Specific detectivity derived from noise spectral density using $D = R A^{1/2} i_n^{-1}$ is marked with *. Specific detectivity values calculated using $D = R (2qJ_D)^{-1/2}$ (assuming dominant shot noise) are reported with †.

Table S2. The series and recombination resistance (R_{sis} and R_{rec}) of the control and TAH devices.

	Rsis (Ω)	Rrec (Ω)
Control	12.3	260
TAH	11.6	346

Reference

[1] W. Wang, D. Zhao, F. Zhang, L. Li, M. Du, C. Wang, Y. Yu, Q. Huang, M. Zhang, L. Li, J. Miao, Z. Lou, G. Shen, Y. Fang, Y. Yan, *Advanced Functional Materials* **2017**, 27.

[2] N. Ma, J. Jiang, Y. Zhao, L. He, Y. Ma, H. Wang, L. Zhang, C. Shan, L. Shen, W. Hu, *Nano Energy* **2021**, 86.

[3] R. Ollearo, J. Wang, M. J. Dyson, C. H. L. Weijtens, M. Fattori, B. T. van Gorkom, A. van Breemen, S. C. J. Meskers, R. A. J. Janssen, G. H. Gelinck, *Nat Commun* **2021**, 12, 7277.

[4] A. Morteza Najarian, M. Vafaie, A. Johnston, T. Zhu, M. Wei, M. I. Saidaminov, Y. Hou, S. Hoogland, F. P. García de Arquer, E. H. Sargent, *Nature Electronics* **2022**.

[5] C. Li, H. Wang, F. Wang, T. Li, M. Xu, H. Wang, Z. Wang, X. Zhan, W. Hu, L. Shen, *Light Sci Appl* **2020**, 9, 31.

[6] Z. Tang, Z. Ma, A. Sanchez-Diaz, S. Ullbrich, Y. Liu, B. Siegmund, A. Mischok, K. Leo, M. Campoy-Quiles, W. Li, K. Vandewal, *Adv Mater* **2017**, 29.

[7] J. Y. Kim, V. Adinolfi, B. R. Sutherland, O. Voznyy, S. J. Kwon, T. W. Kim, J. Kim, H. Ihee, K. Kemp, M. Adachi, M. Yuan, I. Kramer, D. Zhitomirsky, S. Hoogland, E. H. Sargent, *Nat Commun* **2015**, 6, 7772.
